# Supplementary material for: Amenity counts significantly improve water consumption predictions
Source: PLoS One. 2022 Mar 18;17(3):e0265771. doi: 10.1371/journal.pone.0265771 (PMC8932610; doi:10.1371/journal.pone.0265771)
Supplement: S1 Appendix — Links to raw and processed datasets used in this work. (PDF) [file pone.0265771.s001.pdf]

S1 Appendix

Datasets

**Dataset**	**Link**
Population	Facebook High Resolution https://data.humdata.org/dataset/philippines-high-resolution-population-density-maps-demographic-estimates
Elevation	NASA Shuttle Radar Topographic Mission (SRTM) http://e4ftl01.cr.usgs.gov/MEASURES/SRTMGL1.003/2000.02.11/N14E121.SRTMGL1.hgt.zip, accessed through https://dwtkns.com/srtm30m/
Amenity	Full OSM edit history may be downloaded at https://osm-internal.download.geofabrik.de/asia/philippines.html. OSM time filter (https://docs.osmcode.org/osmium/latest/osmium-time-filter.html) was then used to filter by date to produce snapshots of the OSM data.
Water Consumtion Data	Data provided by Manila Water Company, Inc. (MWCI). Processed data can be found at https://github.com/access-at-aim/amenity-water-consumption and is already combined with the population, elevation, and amenity data
Quarantine dates	Gathered from various news releases in the Philippines. Summarized data may be found at https://github.com/access-at-aim/amenity-water-consumption
