# Supplementary material for: Amenity counts significantly improve water consumption predictions
Source: PLoS One. 2022 Mar 18;17(3):e0265771. doi: 10.1371/journal.pone.0265771 (PMC8932610; doi:10.1371/journal.pone.0265771)
Supplement: Attachment — Submitted filename: rebuttal_letter.pdf [file pone.0265771.s002.pdf]

Response to the Editor:

Comment A.1

Please ensure that your manuscript meets PLOS ONE's style requirements, including those for file naming. The PLOS ONE style templates can be found at

https://journals.plos.org/plosone/s/file?id=wjVg/PLOSOne_formatting_sample_main_body.pdf and https://journals.plos.org/plosone/s/file?id=ba62/PLOSOne_formatting_sample_title_authors_affiliations.pdf

Reply and Action A.1

We thank the editor for these resources. After checking, we have found additional style requirements that we missed in PLOS's latex template. We have:

1. Added corresponding author's initials beside email address.
2. Removed funding information from the Acknowledgements section.

Comment A.2

We note that the grant information you provided in the 'Funding Information' and 'Financial Disclosure' sections do not match.

When you resubmit, please ensure that you provide the correct grant numbers for the awards you received for your study in the 'Funding Information' section.

Reply and Action A.2

Thank you for pointing this out. We will ensure that we provide the correct grant numbers (previously stated in our acknowledgments) in the Funding Information.

Comment A.3

Thank you for stating the following in the Acknowledgments Section of your manuscript:

> *"The authors acknowledge the Manila Water Company, Inc. through Jon Michael H. Esteban for providing data used in this work. We also acknowledge John Peter Antonio, Michael Dorosan, Sebastian Ibañez, and Leodegario Lorenzo II for fruitful discussions. This research is primarily funded/monitored by the Department of Science and Technology (DOST) of the Philippines with Project No. 8419, under the CRADLE Program."*

We note that you have provided funding information that is not currently declared in your Funding Statement. However, funding information should not appear in the Acknowledgments section or other areas of your manuscript. We will only publish funding information present in the Funding Statement section of the online submission form.

Please remove any funding-related text from the manuscript and let us know how you would like to update your Funding Statement. Currently, your Funding Statement reads as follows:

> *"The funders had no role in study design, data collection and analysis, decision to publish, or preparation of the manuscript."*

Please include your amended statements within your cover letter; we will change the online submission form on your behalf.

Reply and Action A.3

In line with Comment A.1, we have removed the funding information in our Acknowledgements section. We will also include the funding details in our funding statement upon resubmission. We propose the following amendment to the funding statement:

> *"DD, ML, CA, and CM acknowledge the Department of Science and Technology (DOST) of the Philippines with Project No. 8419, 2020 under the **Collaborative R&D to Leverage the Economy (CRADLE)** Program (https://s4cp.dost.gov.ph/programs/cradle/). The funders had no role in study design, data collection and analysis, decision to publish, or preparation of the manuscript."*

Comment A.4

We note that you have stated that you will provide repository information for your data at acceptance. Should your manuscript be accepted for publication, we will hold it until you provide the relevant accession numbers or DOIs necessary to access your data. If you wish to make changes to your Data Availability statement, please describe these changes in your cover letter and we will update your Data Availability statement to reflect the information you provide.

Reply and Action A.4

We thank the reviewer for this reminder. We are changing our Data Availability statement to

> *All preprocessed data files are available from https://github.com/access-at-aim/amenity-water-consumption.*

We have also mentioned this in our cover letter.

Comment A.5

We note that Figure 1 in your submission contain map image which may be copyrighted. All PLOS content is published under the Creative Commons Attribution License (CC BY 4.0), which means that the manuscript, images, and Supporting Information files will be freely available online, and any third party is permitted to access, download, copy, distribute, and use these materials in any way, even commercially, with proper attribution. For these reasons, we cannot publish previously copyrighted maps or satellite images created using proprietary data, such as Google software (Google Maps, Street

View, and Earth). For more information, see our copyright guidelines: http://journals.plos.org/plosone/s/licenses-and-copyright.

We require you to either (1) present written permission from the copyright holder to publish these figures specifically under the CC BY 4.0 license, or (2) remove the figures from your submission:

a. You may seek permission from the original copyright holder of Figure 1 to publish the content specifically under the CC BY 4.0 license.

We recommend that you contact the original copyright holder with the Content Permission Form (http://journals.plos.org/plosone/s/file?id=7c09/content-permission-form.pdf) and the following text:

> *"I request permission for the open-access journal PLOS ONE to publish XXX under the Creative Commons Attribution License (CCAL) CC BY 4.0 (http://creativecommons.org/licenses/by/4.0/). Please be aware that this license allows unrestricted use and distribution, even commercially, by third parties. Please reply and provide explicit written permission to publish XXX under a CC BY license and complete the attached form."*

Please upload the completed Content Permission Form or other proof of granted permissions as an ""Other"" file with your submission.

In the figure caption of the copyrighted figure, please include the following text: "Reprinted from [ref] under a CC BY license, with permission from [name of publisher], original copyright [original copyright year]."

b. If you are unable to obtain permission from the original copyright holder to publish these figures under the CC BY 4.0 license or if the copyright holder's requirements are incompatible with the CC BY 4.0 license, please either i) remove the figure or ii) supply a replacement figure that complies with the CC BY 4.0 license. Please check copyright information on all replacement figures and update the figure caption with source information. If applicable, please specify in the figure caption text when a figure is similar but not identical to the original image and is therefore for illustrative purposes only.

Reply and Action A.5

We have sent the request letter to stamen design for compliance, and in the meanwhile, we will remove the map backgrounds from our figures. We want to raise specific points of contention however:

1. PLOS One's copyright and license page only ever mentioned that copyrighted content would be under the **CC BY** license, to which we have complied prior to submission.
2. Stamen maps are under the **CC BY license 3.0**. (see http://maps.stamen.com/).

How to Use These Tiles Elsewhere

Except otherwise noted, each of these map tile sets are © Stamen Design, under a Creative Commons Attribution (CC BY 3.0) license.

We'd love to see these maps used around the web, so we've included some brief instructions to help you use them in the mapping system of your choice. These maps are available free of charge. If you use these tiles, you must use this attribution:

Attribution: For Toner and Terrain: Map tiles by Stamen Design, under CC BY 3.0. Data by OpenStreetMap, under ODbL.

< COPY HTML >

3. We opt to remove the stamen map tiles and stick with the default OSM tiles. OSM tiles **should** be usable in our work, as they are under the Open Data Commons Open Database License (ODbL) (https://www.openstreetmap.org/copyright).

Response to Reviewers:

Reviewer #1

Comment 1.1

The introduction section needs further elaboration on the main case study importance, available literature, and the selection of those ML models.

Reply 1.1

We thank the reviewer for the suggestions. We have substantially modified our **Introduction** to further elaborate on the importance of the case study. We have also added discussion on the selection of ML models used in this work.

Action 1.1

About five paragraphs are modified in the Introduction section, highlighting the results of eight newly added references.

Comment 1.2

Case study and data description requires more essential information for better understanding of the readers

Reply 1.2

We thank the referee for this comment. Upon reviewing the manuscript, we agree that additional context on how the water consumption data was obtained would benefit the readers of this work. Our partner Manila Water Company, Inc. (MWCI), provided the Data. They provided us with monthly meter readings of their residential and commercial customers, aggregated at the DMA level. The monthly frequency is a technical limitation, as MWCI lacks AMR devices for higher resolution measurements. While this Data is not public, we will also provide the processed data in a repository.

Action 1.2

We added the following paragraph at the start of the **Water Consumption** (formerly **Data Preprocessing**) section to provide additional context for the water consumption data:

> *Water consumption data were obtained from meter readings of MWCI customers, which include both residential and commercial accounts. Meters were read manually at a monthly frequency due to the lack of AMR devices. These readings are then aggregated at the DMA level and provided by MWCI. Since MWCI also tags their customer accounts as business or residential, they also provided us with the domestic and non-domestic consumption ratio aggregated at the DMA level.*

Comment 1.3

The methodology section needs serious revision. Each models must be reported in more details. Modeling framework required flowchart elaboration. Mathematical concept must be there and add proper citation for the methodology section.

Reply and Action 1.3

We follow this suggestion closely. Mathematical details were initially omitted for brevity, but we agree that additional details help make the paper self-contained. We have revised this section, and here are a summary of the changes:

1. We have renamed the "**Data**" section to "**Methods**"
2. We renamed the "**Features**" section to "**Data**" and placed it under "**Methods.**" We have renamed "**Data preprocessing**" to "**Water Consumption**" and moved it as a subsection of "**Features.**" Additionally, this was expounded to cover the nuances of data collection and the % domestic consumption and # of Quarantine days, which were previously first mentioned in the "**Results and Discussion**" section. We are also moving Table 3 (table of features used in this work) to this section.
3. We have also moved the brief discussion of the Quarantine (which was in the **Results and Discussion**) to the classifications to the **Quarantine Levels** subsection of **Data**.
4. We have expounded on the mathematical details for the three models used: RF, GBT, and SVR as shown in the **Models** section.
5. Additionally, we have reorganized the Models section only to contain two subsections: **Tree-based ensemble methods**, and **SVR**. We are moving **Feature Scaling**, **Model Selection and Tuning**, and **Scoring** to another section named **Machine Learning**
6. We have renamed **Feature Scaling** to **Feature Scaling and Encoding**. We then also added One-Hot Encoding, which was used but not discussed in our previous Methodology.

   … respectively, and One-hot encoding (OHE) \cite{hackeling2017mastering} for categorical data.

   For a random categorical value $\mathbf{s}$ with n possible distinct values $s_1, s_2, …, s_n$, the One-hot encoding of a particular value s_i is a vector $\mathbf{v}$ where every component $ {v}_{i\neq j}=0$ and ${v}_{i=j}=1$. Water consumption was log-transformed as we had previously removed all negative and zero readings. Date features (year and month) were transformed using OHE, as their integer representations'increasing/decreasing progression of does not necessarily correlate with their impact on the model. All other input features were rescaled using min-max scalers.

7. We have added an introductory text to the **Machine Learning** section, which should serve as the modeling framework workflow.

   This section details the machine learning workflow used in this work. Each of the models in the Models section goes through three steps in our workflow. First, the input data undergoes Feature Scaling and Encoding as a preprocessing step. The data and model then undergo Model Selection and Tuning, which trains the model using

the Data while finding the optimal set of hyperparameters to produce the best results. To qualitatively compare models, we need Scoring metrics to give us an objective comparison of the model performance.

Comment 1.4

Figure 4 and 6 are importance and you need to further elaborate and discuss the attained results using the applied algorithm.

Reply 1.4

Thank you. We have added additional discussion on the Feature importance results.

Action 1.4

The sixth paragraph of **Predicting consumption of DMAs** section:

> *Likewise, the minimum and maximum elevations within a DMA may also influence water consumption; elevated areas are usually the most affected when service interruptions occur.*

And at the end of the 7th paragraph, we have added:

> *Apart from Leisure, these establishments serve as robust attractors to human activity, making them good predictors of an area's water consumption. On the other hand, establishments with low SHAP values are more niche to an area, with a couple of notable exceptions. Transport establishments strongly correlate with human movement, but these may not serve as good predictors for consumption as these may capture the transient aspect of mobility. Accommodation includes residential dwellings, and while having the highest amenity count, did not have a high feature importance.*

Reviewer #2

Comment 2.1

In abstract, What is MAE?

Reply 2.1

We thank the referee for pointing out this abiguity. MAE here refers to Mean Absolute Error, which should be defined before the occurrence of this acronym in the abstract.

Action 2.1

We have revised the sentence in the abstract to define MAE:

> *Results show that amenity counts reduce the Mean Absolute Error (MAE) of predictions by 1,440m^3/month or as much as 5.73% compared to just using population and topology features.*

Comment 2.2

please talk about Machine learning in introduction (1 paragraph).

Reply 2.2

We thank the reviewer for the suggestion. We agree that we need to add more discussion on the use of machine learning algorithms in forecasting and the rationale of our chosen models.

Action 2.2

As detailed in Action 1.1, we expounded paragraph 6 of the **Introduction** to include the rationale for selecting the ML models used in this study on top of the initial discussion of machine learning (ML) and deep learning models used in several works related to forecasting.

Comment 2.3

please insert a link for each data you used in the body of the manuscript.

Reply 2.3

Attributions and links to the data sources (Topology, Amenity, Population) used in the manuscript are already provided in the reference section.

Action 2.3

We have added this sentence to the first paragraph of the **Data** section:

> *Links to raw and preprocessed data files are provided S1 Appendix.*

Comment 2.4

L65: what is the resolution of the population data.

Reply 2.4

Details of the population data are described in greater detail in the **Features** section. We mention in L96 that the data has a resolution of 30mx30m.

Action 2.4

We have decided to replace "high-resolution" in L65 to "30m x 30m" to be more upfront with the resolution of the population data. The updated text now reads:

> *(based on 30m x 30m population data [25] and the DMA shapefiles)*

Comment 2.5

Please insert a spatial map contains the population data.

Reply 2.5

Here are two spatial maps of the population data. The first one is the disaggregated raster map of the population (HRSL from Facebook-CIESIN) overlaid on the DMA shapes. In this format, it can be challenging to see the important aspects of the population used in this study, which is the total population at the DMA level.

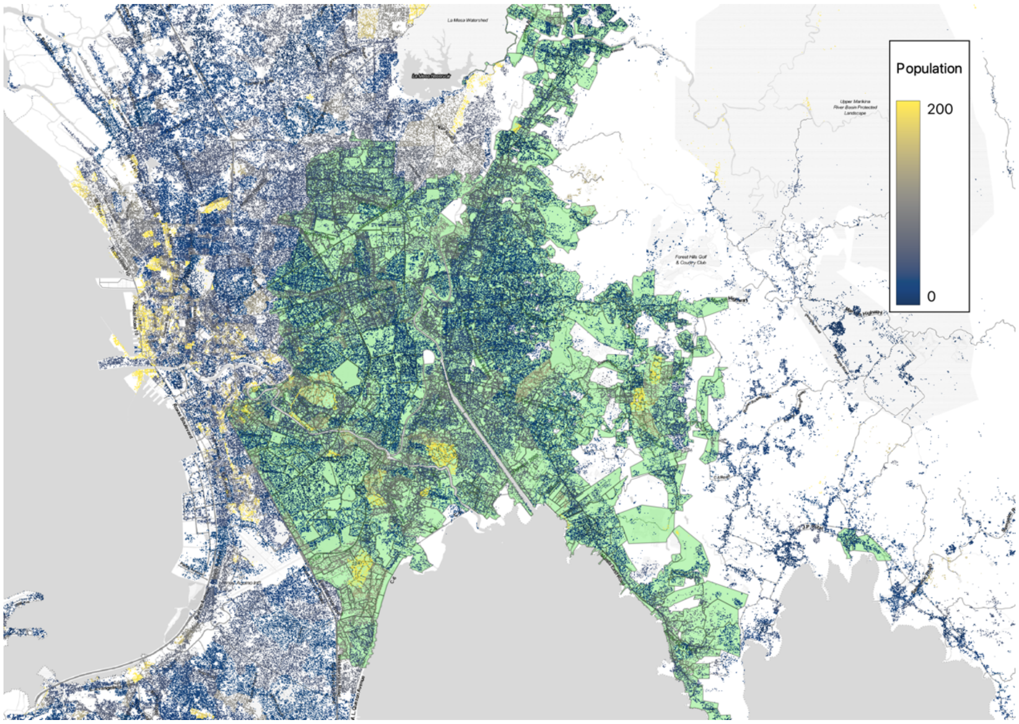

To address this, we aggregated the population counts that fall inside each DMA's shape boundaries to obtain the total population in each DMA. This is the number we ultimately use in our models, and is presented in the next Figure:

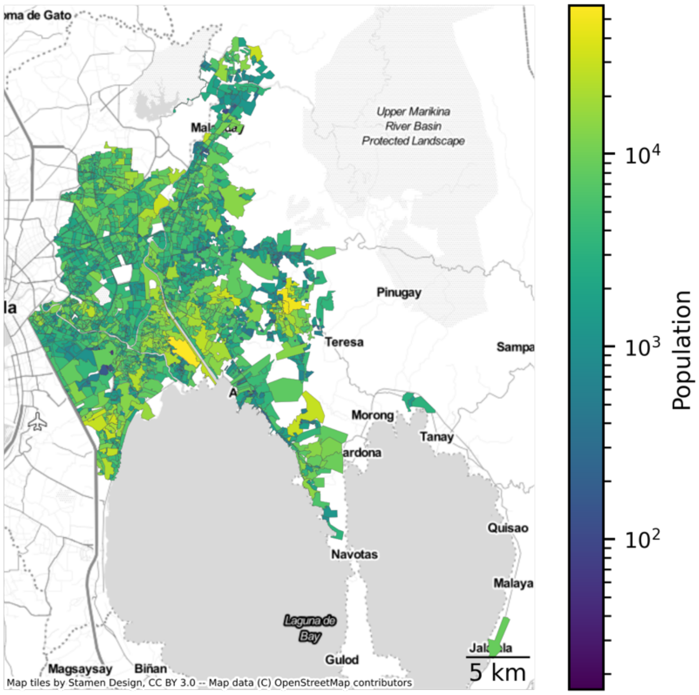

Action 2.5

We have revised the Population section to read as follows:

> *While census data would have been the ideal basis for population data, we faced the limitation that DMAs do not coincide with the resolution of the census (barangay-level, the smallest administrative division). As a workaround, we merged high-resolution population data and the DMA shapefiles to calculate the population inside each DMA (Fig 3). Population data was derived from Facebook's* Data for Good *High-Resolution Settlement Layer (HRSL), which has a spatial resolution of $30 x30m^2$. We used the October 10, 2018 dataset for the population counts.*

We have also added Figure 3: the population map.

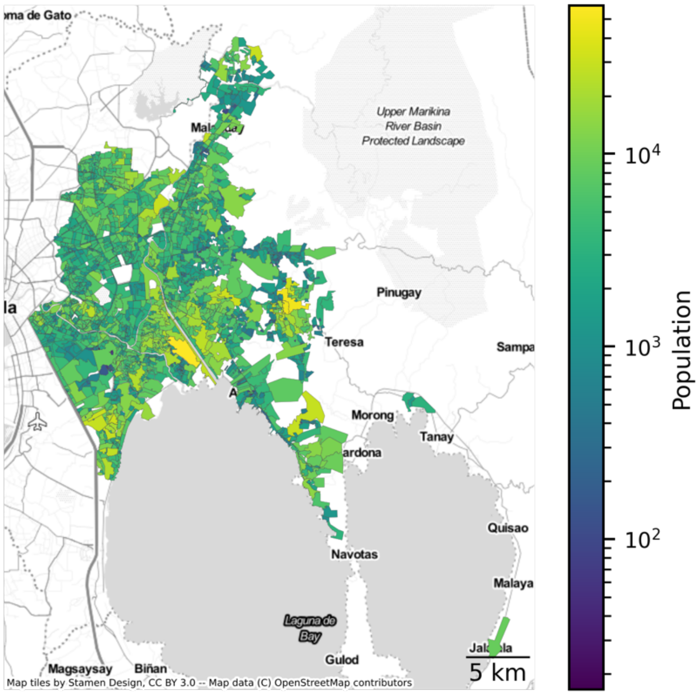

Fig 3. Population data aggregated at the DMA level. *The DMAs cover 371 km^2 and cater to a resident population of around 7.5 million.*

Comment 2.6

Please use Kling-Gupta Efficiency (KGE)with other metrics you used as it evaluate three statistical characteristics together (e.g., Pearson's correlation (r), spatial variability ratio and the normalized variance). You can see more details about it in:

"Hamed MM, Nashwan MS, Shahid S, Ismail T bin, Wang X, Dewan A, et al. Inconsistency in historical simulations and future projections of temperature and rainfall: A comparison of CMIP5 and CMIP6 models over Southeast Asia. Atmos Res. 2022;265: 105927. doi:10.1016/j.atmosres.2021.105927"

"Hamed MM, Nashwan MS, Shahid S. Inter-comparison of Historical Simulation and Future Projection of Rainfall and Temperature by CMIP5 and CMIP6 GCMs Over Egypt. Int J Climatol. 2022;n/a. doi:10.1002/joc.7468".

Reply 2.6

We thank the reviewer for relaying the KGE metric that considers three statistical characteristics as one measure. As a commonly used metric for hydrological studies, this improves the manuscript and increases our reach to domain experts in this field. The inclusion of this metric has also strengthened our claim that Amenity counts contribute significantly to the prediction of water consumption compared to our baseline features, and even reaches KGE>0.5 for the best combination of features (base + (A+D) for DMA consumption, and base+(A+D) and base+(A+Q+D) for the covid Data).

Action 2.6

We have added the KGE as a metric in the Scoring section:

> *We evaluate the results using four prediction accuracy measures: mean absolute error (MAE), mean absolute percentage error (MAPE), the coefficient of determination (R^2), and the Kling-Gupta Efficiency (KGE) \cite{gupta2009}….*
>
> *The KGE is a metric commonly used in evaluating hydrology models \cite{hamed2021, hamed2022, knoben2019} that combines three statistical characteristics: Pearson's correlation r, variability bias, and mean bias. KGE values range from $(-\infty,1]$ with ${KGE}=1$ indicating a perfect fit. While no specific meaning is associated with $ {KGE}=0$, Knoben et al. \cite{knoben2019} argues that values of $ {KGE}>1-\sqrt{2}$ indicate that the model prediction is better than the mean, even if the $ {KGE}$ value is negative.*

Additionally, we included the KGE metric in the results and discussion in several places for the **Predicting consumption of DMAs** section, middle of the 3rd paragraph:

> *The KGE metric further supports this observation, showing amenities clearly outperform the base set, with the correlation, mean, and variance taken into account.*

And at the end of the 4th paragraph:

> *All metrics point to the case of base + (A+D) as the best prediction model. However, the ratio of domestic to non-domestic consumption comes from MWCI's classification of customer accounts, something that might not be available when predicting consumption of currently unserved or underserved areas.*

We also added this to the discussion in the **COVID-19 pandemic disruptions and quarantine feature labels** section, 2nd paragraph:

> *With the inclusion of amenities as features, even with the disruption due to the pandemic, implementations of GBR and LGBM perform the best among the MAE, MAPE, R^2, and KGE metrics.*

Comment 2.7

Please indicate the software you used in the analysis.

Reply and Action 2.7

We used Python and the packages mentioned in the manuscript (scikit-learn and lightgbm) to implement the machine learning models. We have made these more explicit in our revised Models section in our Methodology to address the comments of Reviewer 1. Specifically, we indicated the exact function implementations within the sklearn and lightgbm packages used per type of model.

Comment 2.8

Please separate the results and discussion.

Reply and Action 2.8

We thank the reviewer for this comment. Given that our work presents three main results, we believe that separating the discussion reduces the readability of this section's overall narrative and organization, placing the interpretations too far away from the specific results it refers to. However, the reviewer's comment allowed us to see points for improvement in our narrative; we rearranged the presentation of results by placing the data analysis of water consumption patterns as the first part of the section, followed by the two machine learning model results. We also added a brief introduction to this section to guide the reader into the main highlights of the work they should expect.

> *This section is divided into the three highlights of this work. First, we present an exploratory data analyses on representative DMAs and examine the changes in consumption patterns due to the COVID pandemic. We then present our model for predicting water consumption of DMAs using machine learning. Lastly, we look at the robustness of our model in predicting water consumption when population movement is reduced with the inclusion of pandemic restrictions as features.*

We also moved some parts like Table 3 *(Description of all features used in this work)* to the **Methods** where it was more aligned. This also made the Results and Discussion more organized and, hopefully, easier to read.

Comment 2.9

First paragraph in results and discussion should be in the introduction.

Reply 2.9

We thank the reviewer for this suggestion. We note that the said paragraph was initially discussed in the **Introduction** (Paragraph 4) and was placed again in the **Results and Discussion** section to give a brief recap of the motivation of this work. We do acknowledge that in its current form, it looks out of place in the **Results and Discussion**, thus we have decided to make it more concise and omit the statistics previously mentioned in the **Introduction**.

Action 2.9

The said paragraph in the **Results and Discussion** section was made more concise. It is now located at the first paragraph of the **Predicting consumption of DMAs**, which was moved down because of our restructured section (see Comment 2.8).

> *A high percentage of the Philippine population still lacks piped water connections to their homes. Since government regulation caps profits from utilities, water utility companies have a strong incentive to expand their coverage to generate revenue. Water consumption predictions for a new DMA can help potential water utility companies plan additional water lines to serve these areas while minimizing potential losses due to mismatched supply and demand.*
